# Modulation of gut microbiota through nutritional interventions in Behçet’s syndrome patients (the MAMBA study): study protocol for a randomized controlled trial

**DOI:** 10.1186/s13063-020-04444-6

**Published:** 2020-06-09

**Authors:** Giuditta Pagliai, Monica Dinu, Claudia Fiorillo, Matteo Becatti, Silvia Turroni, Giacomo Emmi, Francesco Sofi

**Affiliations:** 1 Department of Experimental and Clinical Medicine, School of Human Health Sciences, University of Florence, Largo Brambilla 3, 50134 Florence, Italy; 2 Unit of Clinical Nutrition, Careggi University Hospital, Florence, Italy; 3 Department of Experimental and Clinical Biomedical Sciences “Mario Serio”, University of Florence, Florence, Italy; 4 Department of Pharmacy and Biotechnology, Unit of Holobiont Microbiome and Microbiome Engineering, University of Bologna, Bologna, Italy; 5 Interdisciplinary Internal Medicine, Center for Autoimmune Systemic Diseases, Behçet Center and Lupus Clinic, Careggi University Hospital, Florence, Italy; 6 Don Carlo Gnocchi Foundation Italy, Onlus IRCCS, Florence, Italy

**Keywords:** Gut microbiota, Mediterranean diet, Vegetarian diet, Short-chain fatty acids

## Abstract

**Background:**

Behçet’s syndrome (BS) is a systemic inflammatory disorder of unknown etiology, and it is characterized by a wide range of potential clinical manifestations. Recent evidence suggests that the gut microbiota (GM) in BS has low biodiversity with a significant depletion in butyrate producers. The aim of the present project is to investigate whether a dietary intervention could ameliorate the clinical manifestations and modulate the GM of individuals with BS.

**Methods:**

This is a randomized, open, cross-over study that involves 90 individuals with BS, who will be randomly assigned to one of three different diets for 3 months: a lacto-ovo-vegetarian diet (VD), a Mediterranean diet (MD), or a Mediterranean diet supplemented with butyrate (MD-Bt). The VD will contain inulin-resistant and resistant-starch-rich foods, eggs, and dairy in addition to plant-based food, but it will not contain meat, poultry, or fish. The MD will contain all food categories and will provide two portions per week of fish and three portions per week of fresh and processed meat. The MD-Bt will be similar to the MD but supplemented with 1.8 g/day of oral butyrate. The three different diets will be isocaloric and related to the participants’ nutritional requirements. Anthropometric measurements, body composition, blood, and fecal samples will be obtained from each participant at the beginning and the end of each intervention phase. The primary outcomes will be represented by the change from baseline of the BS gastrointestinal and systemic symptoms. Changes from baseline in GM composition, short-chain fatty acid (SCFA) production, and the inflammatory and antioxidant profile will be considered as secondary outcomes.

**Discussion:**

BS is a rare disease, and, actually, not all the available treatments are target therapies. A supportive treatment based on dietary and lifestyle issues, able to restore immune system homeostasis, could have a high impact on cost sustainability for the treatment of such a chronic and disabling inflammatory condition.

**Trial registration:**

clinicaltrials.gov: NCT03962335. Registered on 21 May 2019.

## Introduction

Behçet’s syndrome (BS) is a systemic inflammatory disorder characterized by a wide range of potential clinical manifestations with no gold-standard therapy [1, 2]. Although the pathogenesis of BS is currently unknown, it has been recently classified as a type of systemic vasculitis [3]. The gut microbiota (GM) is recognized to deeply influence our metabolic and immunological health, and specific dysbiotic GM configurations indicate a fascinating link between intestinal microbes and health status. A recent study from our group [4] showed, for the first time, that a peculiar dysbiosis of the GM is present also in individuals with BS, in line with several other chronic disorders [5–7]. Moreover, individuals with BS have significant depletion of well-known butyrate producers, i.e. *Roseburia* and *Subdoligranulum*, and a consistent decrease in butyrate, one of the most representative short-chain fatty acids (SCFAs), thus suggesting an increase in the inflammatory state [8, 9].

In this context, over recent years, growing evidence has suggested that high-fiber dietary patterns promote a more favorable GM profile, and are key mediators of microbial diversity [10, 11]. Lacto-ovo-vegetarian diet is characterized by abstention from consuming meat and meat products, poultry, seafood, and flesh from any other animal and by consuming a large amount of plant-derived foods. This dietary pattern has been largely demonstrated to be beneficial both in patients with established disease and in people with with traditional risk factors for chronic diseases [12, 13]. In particular, it has recently been demonstrated that high adherence to a lacto-ovo-vegetarian diet is associated with a beneficial GM profile, with enrichment in fiber-degrading bacteria and an increase in fecal SCFAs [14]. Similarly, other dietary patterns rich in plant-based food, such as the Mediterranean diet, have been shown to modulate GM dysbiosis, by supporting the recovery of a balanced microbial community of health-promoting SCFA-producing members with the decrease in pro-inflammatory pathobiont groups [15, 16]. Furthermore, current evidence indicates that the consumption of certain fibers - such as inulin and resistant starch - leads to specific GM rearrangements with consequent butyrate hyperproduction in humans [17]. All these findings let us hypothesize that the adherence to a controlled dietary profile enriched in substrates with potential for butyrate production may select for butyrate-producing bacteria - such as *Roseburia* spp. and *Faecalibacterium prausnitzii* - so reversing the pro-inflammatory dysbiosis observed in BS.

Thus, the aim of the present project is to conduct an open, randomized, cross-over dietary intervention trial to investigate whether a lacto-ovo-vegetarian diet enriched in substrates with potential for butyrate production or a Mediterranean diet supplemented with butyrate could be beneficial for the GM and the amelioration of the clinical manifestations and disease severity in individuals with BS.

## Methods: participants, interventions, and outcomes

### Study design

**Fig. 1:**
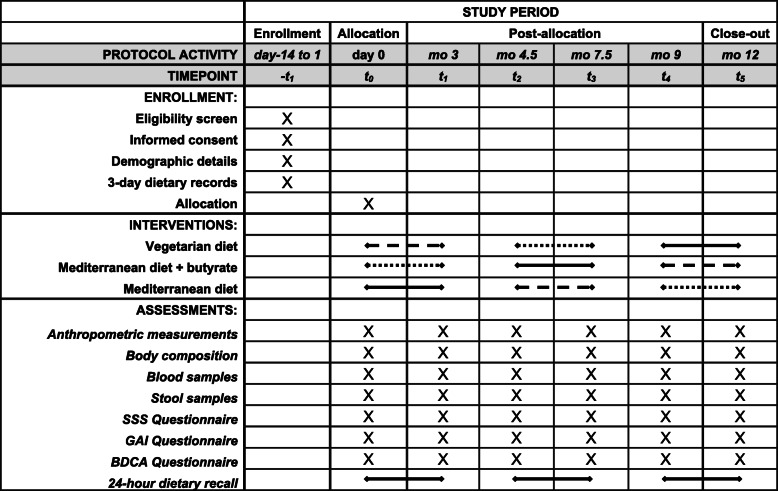
Schedule of enrollment, interventions, and assessments for participants. SSS, Symptom Severity Scale; GAI, Global Assessment of Improvement Scale; BDCA, Behçet Disease Current Activity Form; mo, month

The randomized, open, cross-over clinical trial will be conducted at the Careggi University Hospital, Florence, Italy. A cross-over design will be implemented to allow comparison of a lacto-ovo-vegetarian diet (VD), a Mediterranean diet supplemented with butyrate (MD-Bt), and a Mediterranean diet without any supplement (MD), as control, within the same individual. Participants will act as their own controls in cross-over studies, so individual differences will be controlled for, making the error variance smaller and subsequently reducing the sample size required to find a significant effect due to increased statistical power. The study design follows the Standard protocol items: recommendation for interventional trials (SPIRIT) guidelines (see Fig. 1 and supplementary file #1).

### Eligibility criteria

Inclusion criteria include diagnosis of BS, age 18–65 years, willing to give informed consent, and willing to participate in a study where one of the proposed diets has a vegetarian pattern.

Exclusion criteria include pregnancy or lactation; concomitant presence of serious illness or unstable condition (other immune-mediated or autoimmune diseases, including inflammatory bowel diseases); chronic viral infections; malignancies; recent myocardial infarction; chronic liver disease; current or recent (past 6 months) participation in a weight loss treatment program or use of weight loss medication; adoption of a vegetarian diet for the past 3 months; antibiotic, prebiotic, or probiotic use in the past 3 months; and current or previous (past 6 months) infection with SARS-CoV-2.

### Interventions and participant timeline

This clinical randomized study has a cross-over design with three intervention periods and two wash-out periods. After a 2-week run-in period - which will be used to assess participants’ eligibility and to collect demographic details, signed informed consent, and 3-day dietary records - the eligible participants (*N* = 90) will be randomly assigned to follow a 3-month dietary profile with a VD, a MD or a MD-Bt. The VD will contain inulin and resistant-starch-rich foods, eggs, and dairy, in addition to plant-based food, but will not contain meat, poultry, or fish. The MD will contain all the food categories and will provide two portions per week of fish and three portions per week of fresh and processed meat (one of which will consist of fresh or processed red meat). The MD-Bt will be similar to the MD but supplemented with 1.8 g/day of oral butyrate. The three different dietary patterns will be isocaloric and related to the participants’ nutritional requirements, with about 50–55% of energy derived from carbohydrates, < 30% from fats, and 15–20% from proteins. Participants will prepare their meals or eat at restaurants. Alcoholic beverages will be limited to two per day for men and one per day for women. Interventions will be delivered by a dietitian through face-to-face, individual counseling sessions at the Careggi University Hospital. Participants will be provided with a detailed, 1-week menu plan with portions expressed in grams or milliliters as appropriate, and tips and information on the food groups that can be included and those that cannot. The VD plan will also include recipes for preparing meals.

**Fig. 2:**
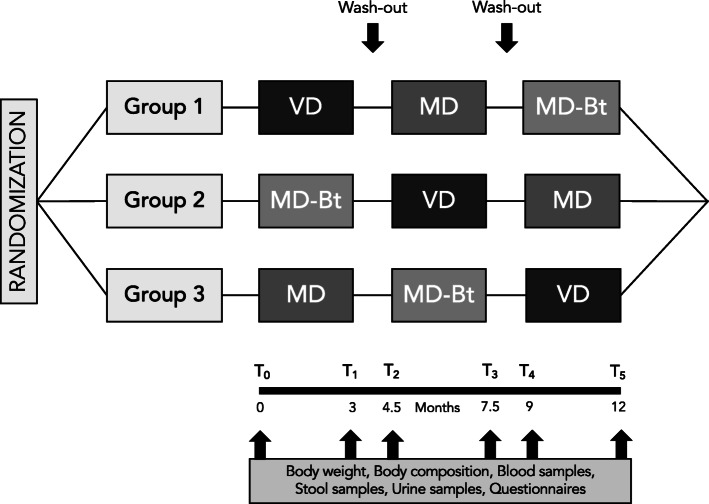
Study design. VD, vegetarian diet; MD, Mediterranean diet; MD-Bt, Mediterranean diet supplemented with butyrate

The study design is depicted in Fig. 2. There will be six clinical evaluations of the study population: at baseline before starting the nutritional interventions (T0), 3 months after the onset of the first nutritional intervention (T1), at the end of the first wash-out period, lasting 1.5 months, when individuals will be allowed to resume their normal eating habits, at the onset of the second intervention (T2) at 7.5 months after the onset of the study, at the end of the second nutritional intervention (T3), at the beginning of the third nutritional intervention and at the end of the wash-out period, lasting again 1.5 months (T4), and at the end of the third nutritional intervention (T5).

During the baseline visit, participants will be instructed on the objectives and methods of the clinical trial and will be asked not to alter their physical exercise habits during the study. Anthropometric measurements, body composition, and blood, urine, and stool samples will be obtained from each participant at the beginning and the end of each intervention phase. The clinical manifestations and disease severity of BS, especially symptoms involving the gastrointestinal system, will be assessed using a modified version of two questionnaires originally computed for individuals with inflammatory bowel disease: the Global Assessment of Improvement Scale (GAI) and the Symptom Severity Scale (SSS). Disease activity will be assessed by the validated Behçet Disease Current Activity Form (BDCAF), which consists of objective and subjective items, and considers the symptoms present over the 4 weeks before the assessment. Each participant will have to complete a 3-day dietary record (two weekdays and a weekend day) before starting and a dietician will analyze all 3-day dietary records using a country-specific food-nutrient database.

### Outcome measures

#### Primary outcomes

Primary outcomes will be assessed through validated questionnaires, to analyze the progression of BS. The metric used will be changes in means from the beginning to the end of each dietary intervention. They will include:

- Severity of gastrointestinal symptoms assessed by the SSS modified form. The SSS is a multidimensional rating scale assessing overall symptom severity on a visual analogue scale (VAS). An overall score will be calculated from six items: pain severity, pain frequency, abdominal bloating, dissatisfactory bowel habit, abdominal heaviness, and life interference. The modified SSS ranges from 0 to 600, with higher scores meaning more severe symptoms.
- Improvement of gastrointestinal-related BS symptoms assessed by the GAI modified form. The GAI will assess improvement of symptoms of BS using a 7-point scale, with higher scores meaning an improvement in the symptoms. The severity of abdominal pain, severity of abdominal distention, satisfaction with bowel habits, severity of headache, severity of exhaustion, severity of nausea, attention disorder, muscle/joint pain, and quality of life will be investigated in response to the following question: “Compared to the way you felt before you entered the study, have your symptoms over the past 7 days been: 1) “Substantially Worse”, 2)” Moderately Worse, 3)” Slightly Worse”, 4)” No Change”, 5)” Slightly Improved”, 6)” Moderately Improved” or 7) “Substantially Improved”.
- Disease severity in BS assessed by the BDCAF. The BDCAF will assess the presence of oral and genital ulceration, skin, joint, and gastrointestinal involvement, presence of fatigue and headache, using a 5-point scale according to the duration of symptoms, with 0 meaning no symptoms and 4 meaning symptoms for 4 weeks. The presence of eye, large vessel, or central nervous system (CNS) involvement will be documented with “yes/no” answers. In addition, individuals will be asked to rate on a 7-point scale how active they felt. Similarly, clinicians will complete a 7-point rating scale to assess their opinion of overall disease activity, with lower scores representing better outcomes.

#### Secondary outcomes

Secondary outcomes will be measured in blood, urine, and stool samples. The metric used will be changes in means from the beginning to the end of each dietary intervention. Stool samples will be analyzed to assess:

- Changes from baseline in the composition of the GM, assessed by 16S rRNA gene-based next-generation sequencing on the Illumina MiSeq platform. Total microbial DNA will be extracted from feces using the repeated bead-beating plus column method, as previously described [18]. The V3 and V4 hypervariable regions of the 16S rRNA gene will be sequenced following the Illumina protocol for 16S Metagenomic Sequencing Library Preparation.
- Change from baseline in fecal SCFAs assessed by gas chromatography - mass spectrometry (GC-MS). The metabolomic analysis of fecal waters will be performed after sample preparation involving solid phase microextraction (SPME), followed by GC-MS analysis to detect the volatile metabolites [4].
- Change from baseline in the fecal inflammatory profile assessed by the cytofluorimetric approach. In particular, superficial (CD3, CD4, CD8) and intracytoplasmic (transforming growth factor (TGF)-ß, interferon gamma (IFN)-γ, interleukin (IL)-4, IL-9, IL-10, IL-17, IL-10, IL-22, FoxP3) cell markers will be analyzed. The cells will be analyzed by the BD FACScan Cytofluorimeter using the Diva software (BD Biosciences, San Jose, USA). The concentration of cytokines, chemokines, and growth factors, including interleukins, IFN-γ, tumor necrosis factor (TNF)-α, growth-regulated oncogene-α (Gro-α), monocyte chemoattractant protein-1 (MCP-1), macrophage inflammatory protein-1ß (MIP-1ß), granulocyte colony-stimulating factor (G-CSF), and granulocyte-macrophage colony-stimulating factor (GM-CSF) will be determined in the fecal solutions according to the methodology of Munoz-González et al. [19].

Blood samples will be analyzed to assess:

- Change from baseline in the inflammatory profile assessed by the cytofluorimetric approach. It will be assessed using the Bio-Plex cytokine assay (Bio-Rad Laboratories Inc., Hercules, CA, USA), according to the manufacturer’s instructions. In particular, IL-1ra, IL-4, IL-6, IL-8, IL-10, IL-12, IL-17, MCP-1, MIP-1ß, vascular endothelial growth factor (VEGF), TNF-α, IFN-γ, and IFN-γ-induced protein (IP)-10 will be measured.
- Change from baseline in the lipid peroxidation markers assessed by spectrophotometry. It will be estimated using the thiobarbituric acid reactive substances (TBARS) assay kit [20].
- Change from baseline in plasma total antioxidant capacity (TAC) assessed by fluorometry, using oxygen radical absorbance capacity [21].
- Change from baseline in reactive oxygen species (ROS) assessed by flow cytometry. In particular, leukocyte subpopulations (lymphocyte, monocyte, and granulocyte) ROS will be measured [22].

Urine samples will be analyzed to assess:

- *1,4-dihydroxynonane mercapturic acid (DHN-MA) change from baseline*, assessed by enzyme immunoassay using polyclonal antibodies [23]. This specific biomarker is the major urinary metabolite of 4- hydroxy-2-nonenal, a lipid peroxidation product.

### Sample size calculation

Due to the lack of dietary intervention trials on BS and to the fact that gastrointestinal symptoms are quite similar in BS and inflammatory bowel disease, based on a previously published trial [24], a sample size of 80 individuals with BS is required to achieve power of 80% (beta) with alpha = 0.05, to detect a 50-point difference in the mean of SSS (the primary outcome), between VD and MD interventions. We will recruit an extra 10 volunteers (for a total of 90), as we assume that not all individuals would be compliant with the treatments and in case of loss to follow up. Losses will be included in the intention-to-treat but not in the per-protocol analyses.

### Recruitment and randomization

Participants will be recruited from the Behçet Center and Lupus Clinic, Careggi University Hospital, Florence, Italy, or using advertisements on local media, newspapers, social media and websites. After approval and completion of the initial assessment, the participants will be formally included in the study and randomized 1:1:1 to the three intervention arms through a web-based online randomization procedure. No adaptive randomization procedures will be performed. The random allocation sequence will be produced and managed by an independent staff member who is outside of the project, to code the treatments, and maintain the key to this code until data collection is completed.

### Blinding

Although full blinding of both participants and dieticians to treatments in this study is not possible because of obvious differences between the intervention diets, several strategies will be employed to reduce the risk of bias. First of all, the treatment allocation for each individual will not be revealed until the individual has irrevocably been entered into the trial, to avoid selection bias. In addition, outcome measures in the present study cannot be easily influenced by the observer. Furthermore, trial personnel who will enroll participants, data collectors, outcome assessors, and data analysts will be blinded to treatment allocation, and an employee outside of the research team will insert data into the computer in separate datasheets. On the other hand, making the trial open rather than blinded may improve recruitment. Unblinding will be permissible only when knowledge of the treatment will be absolutely essential for further management of the individual.

### Data collection

Follow-up assessments and data collection will be undertaken at the Unit of Clinical Nutrition of the Careggi University Hospital, Florence, Italy, by trial personnel. All participants will be examined between 7.30 and 11.30 a.m. after a 12 h-fasting period.

#### Compliance

Compliance with the interventions will be achieved using behavior change strategies including self-monitoring, and regular phone calls for dietary counseling. In particular, participants will receive at least one unannounced phone call during each intervention, in which participants will recall his or her 24-h diet period. Furthermore, participants will be provided with a detailed one-week menu plan for each dietary period with all foods expressed in weight and/or volume measures, and a hand-out containing details on their assigned diet, including food groups that can be included and ones that should be avoided. The vegetarian menu plan will also include recipes for preparing meals. Participants may discontinue the intervention or withdraw from the study for the following reasons: (1) at the request of the participant; (2) if the investigator considers that a participant’s health will be compromised due to adverse events or concomitant illness that develop after entering the study. Participants prematurely discontinued from the study before the 3-month evaluation will have the baseline clinical and laboratory evaluations performed.

#### Anthropometric measurements and body composition

Weight and height will be measured using a stadiometer. Body mass index (BMI) will be calculated as weight (kilograms)/height (meters squared). Individuals will be classified as overweight if their BMI is more than 25 kg/m^2^ but less than 30 kg/m^2^, and obese if their BMI is 30 kg/m^2^ or more. Body composition will be determined using a bioelectrical impedance analyzer (TANITA, model BC 420 MA) at the beginning and the end of each intervention phase.

#### Blood samples

Blood samples will be collected at each clinical evaluation. Blood samples will be centrifuged at 3000 rpm for 15 min to yield serum, aliquoted, and then stored at − 80 °C for subsequent analysis.

#### Urine and stool samples

Urine and fecal samples (four or five scoops totaling 4 g) will be collected in sterile containers with no solution before and after each intervention phase - with a total of six samples from each participant - and immediately frozen at − 20 °C, before being transferred to − 80 °C for subsequent analysis. The participants will be provided with urine and stool sample collection kits including containers and instructions. Participants will be invited to place the stool samples in the supplied sterile container with no solution, placed in a plastic bag and put into a household refrigerator immediately. Urine and stool samples will have to be delivered to the laboratory within 24 h in a styrofoam box containing ice packs.

#### Storage of biological specimens

The storage of biological specimens will be performed under appropriate conditions according to standard methods. Blood samples will be aliquoted and stored at − 80 °C for 4 years before being used or destroyed. Stored samples will be used exclusively for research purposes upon the consent of the donor. Sample destruction will be appropriately documented.

### Data management

Data will be collected in an electronic database. Identifiable data or other documents will not be recorded in the database and participants will be identified by a unique trial ID only. Hard copies of data sheets linking the participant identification number to the person’s contact details will be kept securely in a locked filing cabinet in a locked office, accessible only to key research team members. Participant files and other source data (including copies of protocols, questionnaires, original reports of test results, correspondence, records of informed consent, and other documents pertaining to the conduct of the study) will be kept for the maximum period permitted by the institution. All study data will be stored in the DASH-IN infrastructure, which is developed by ENPADASI. Thereby we will adhere to the findable, accessible, interoperable, and reusable (FAIR) principles. The data will be made open access upon publication. Within Europe, the ELIXIR infrastructure is coordinating data stewardship and management activities in the life sciences.

Multiple strategies will be employed to improve data quality during data collection, including accurate recruitment, a structured and time-limited protocol, the inclusion of a run-in period, the limitation of the burden and inconvenience of data collection to the participants, the development of a trusting and collaborative relationship between research units and participants, and double data-entry.

### Statistical analysis

The results will be expressed as mean plus/minus SD, median and range, or geometric mean with 95% confidence intervals (CIs) as appropriate. Categorical variables will be presented as frequencies and percentages. All data will be treated as paired samples from a cross-over study. The three interventions will be analyzed combining the results obtained in the three phases of the three groups. Outcomes will be analyzed within each group using the paired comparison Student’s *t* test to test whether the changes are statistically significant. The absolute change (mean baseline value subtracted from mean value after each intervention) will be estimated using the independent samples *t* test.

Raw sequences will be processed in the GM analysis, using a pipeline that combines PANDAseq [25] and QIIME [26]. The UCLUST software [27] will be used to bin high-quality reads into operational taxonomic units (OTUs) at 0.97 similarity threshold through an open-reference strategy. Taxonomy will be assigned through the Ribosomal Database Project (RDP) classifier, using the Greengenes database as a reference. Alpha diversity and rarefaction curves will be computed using different metrics, including the Faith’s phylogenetic diversity, the number of observed OTUs and the Shannon index. Beta diversity will be estimated by weighted and unweighted UniFrac distances, which will be used as input for principal coordinates analysis (PCoA). All statistical analysis will be performed using R and the packages vegan, stats, and made4. The significance of data separation in the PCoA will be tested using a permutation test with pseudo-F ratios (function Adonis in vegan). The Wilcoxon test for paired data will be used to assess significant differences in alpha diversity and taxon relative abundance between groups, and the Kruskal–Wallis test will be used for multiple comparisons. The *p* values will be corrected for false discovery rate (FDR) using the Benjamini-Hochberg method.

Permutational analysis of variance (PERMANOVA) adjusted for the order of treatment will be applied with two objectives: to assess the effect of the dietary interventions on PCoA scores of beta diversity metrics and to assess the effect of the dietary interventions and several metadata (SCFA levels, nutrient intake, clinical and biochemical parameters) on the whole microbial composition. Finally, the analysis will be performed at each taxonomic level (genus, family, order, class and phylum), in a separate model for each piece of metadata. Each analysis will be performed using a model including both the main effects and the interaction of the three dietary interventions. Because these tests assume normal data distribution, non-distributed data will be transformed into logs, and further analyses will be performed with the processed data. However, to facilitate interpretation, the log data will be again converted to the original scale (antilog) and presented as geometric means with 95% CIs. Spearman correlation coefficients between the changes in microbial composition, SCFA levels, nutrient intake, and in clinical and biochemical parameters will be computed and statistically tested for each dietary intervention.

Before starting the data analysis, the level, pattern and likely causes of the missingness in the baseline variables and outcomes will be investigated by preparing appropriate tables. This information will be used to determine whether the level and type of missing data has the potential to introduce bias into the analysis results or substantially reduce the precision of estimates for the proposed statistical methods. Sensitivity analyses will be undertaken, based on the assumption that missing outcomes are the worst possible, or the best possible, in different randomization groups. If these show that conclusions may differ based on missing values, then supplementary multiple imputations for missing values will be undertaken. These analyses will account for results of any losses to follow up insofar as they pertain to differences in measured variables (i.e. under the assumption of missing at random). Finally, subgroup analyses will be performed to analyze possible differences in the changes according to some characteristics of the study population, such as age, sex, categories of BMI, and physical activity. A *p* value <0.05 will be considered statistically significant. Statistical analyses will be performed using SPSS software for Macintosh (SPSS Inc., Chicago, IL, USA).

### Monitoring

Given the limited objectives and its short-term nature, this trial will be monitored on a regular basis by the protocol team and the local Institutional Review Board, without the use of a formal data monitoring committee. Each month, the protocol team will provide the local Institutional Review Board with a monitoring report, including a review of activities, progress, difficulties, and issues of concern. No ad interim analysis will be performed. Data access will be restricted to trained staff with unique password-protected accounts. Adverse events such as unfavorable and unintended signs, abnormal laboratory findings, symptoms, or diseases temporally associated with the intervention diet will be collected from the time of randomization until the final 12-month follow-up visit for each participant, whether or not considered related to the intervention study. All adverse events will be followed up until they are resolved.

## Discussion

BS is a systemic inflammatory disorder characterized by a wide range of potential clinical manifestations affecting different organs and tissues [1], with higher risk of mortality due to vascular-thrombotic and neurological affections [2]. The etiology remains unknown, and although various mechanisms have been proposed, it is not yet clear whether the microbiome has a role in this process [28]. To date, no studies are available that evaluate the effects of a nutritional intervention on BS. Thus, the aim of the project will be to understand the effect of a VD, MD-Bt, or MD on the manifestations of BS and, possibly, the role of the intestinal microbiota as a mediator of dietary effects in individuals with BS.

In a recent study by our group [4] there was low biodiversity in the composition of the GM in individuals with BS, and specific changes in the profiles of SCFA production [4]. In particular, a significant depletion of butyrate producers - such as *Roseburia* and *Subdoligranulum -* and a consequent decrease in butyrate production was demonstrated. Butyrate is the preferred fuel for colonocytes and one of the most representative SCFAs. It induces T regulatory cell differentiation through several mechanisms, so butyrate impairment in individuals with BS could promote reduced T regulatory cell-mediated control, thus promoting a powerful immunopathological T cell response [8] as suggested by the higher percentage of T helper (Th1)/Th17 cells in the intestinal mucosa observed in BS [9]. The presence of an altered GM in BS has recently been confirmed by Ye et al. [29] by analyzing fecal and saliva samples collected from 32 individuals with BS and 74 healthy controls. Fecal samples from individuals with BS contained greater levels of *Bilophila spp*. and several opportunistic pathogens (i.e. *Parabacteroides spp*. and *Paraprevotella spp*.) together with lower levels of butyrate-producing bacteria *Clostridium spp.* and methanogens (*Methanoculleus spp*. and *Methanomethylophilus spp*.). These differences have been associated with altered biological microbial functions with an enhanced oxidation–reduction process, a capsular polysaccharide transport system, and type III and IV secretion systems [29].

Increased evidence suggests that dietary patterns characterized by increased amounts of plant-based foods, such as a MD or VD, have positive effects on health status, possibly modulating GM and the production of its metabolites [14, 16, 30]. Indeed, dietary patterns rich in non-refined cereals, fruits, vegetables, and legumes have been found to promote a healthier GM profile due to the large amount of dietary fiber. These fermentable substrates are sources of metabolic fuel for the fermentation of GM, which, in turn, results in end products - mainly SCFAs - that are key microbial metabolites with a multifactorial role in host health [13]. In particular, foods rich in inulin i.e. chicory, artichokes, and onions, and foods rich in resistant starch i.e. cooked and chilled rice, pasta, or potatoes, have been associated with increased butyrate production [17]. In this regard, a recent study by our group [30], which compared microbial and metabolic changes after a 3-month dietary intervention with MD and VD, reported a significant positive association of carbohydrate consumption with fecal butyrate levels and a significant negative association of fat intake with propionate and acetate. Negative associations were also observed between SCFA and levels of several inflammatory cytokines.

BS is a rare disease, and the available treatments are not specifically tailored therapies. Beyond the real progress made in the treatment of this disease in the past decade, the management of BS remains a challenge for physicians. Increasing evidence demonstrates that it is a disorder with an extremely wide spectrum of clinical features that can respond to certain specific treatments which, however, can be ineffective on other manifestations or even worsen some features. A treatment based on dietary and lifestyle issues, able to restore immune system homeostasis, could have a high impact on cost sustainability in the treatment of such a chronic and disabling inflammatory condition.

### Trial status

The trial has received all necessary regulatory approvals. The current approved protocol version is 2.0 (version date 16 April 2018). We anticipate a recruitment start date of 16 September 2019 and a recruitment completion date of 15 June 2020. The enrolment of participants with BS is ongoing at the time of manuscript submission.

## Supplementary information

**Additional file 1.** SPIRIT 2013 Checklist: Recommended items to address in a clinical trial protocol and related documents.

**Additional file 2.**

## Data Availability

The results from this clinical trial have the potential for immediate public health applicability. The target audience will be reached through publications, oral presentations, and seminars. Data analysis and manuscript preparation will occur during the last 6 months of this proposed trial. All plans for dissemination of study results will be discussed with the investigators before implementation. Any amendments to the protocol and information provided to participants will be submitted to the Ethics Committee for approval before implementation. Substantial amendments may only be implemented after written approval has been obtained whereas non-substantial amendments can be implemented without written approval from the Ethics Committee. The Chief Investigator will have to ensure that the participant’s privacy is maintained. Data and source documents will be stored in such a way that they can be accessed at a later date for the purposes of monitoring or inspection by the Ethics Committee. At the end of the study, participants will be able to request a copy of the results of the study from the Chief Investigator. The results from the trial will be submitted for publication in a peer-reviewed journal irrespective of the outcome. The final report will follow the CONSORT 2010 guidelines. Authorship of presentations and reports related to the study will be in the name of the collaborative group.

## Abbreviations

BDCA: Behçet disease current activity form
BMI: Body mass index
BS: Behçet’s syndrome
CNS: Central nervous system
FoxP3: Forkhead box P3
GAI: Global Assessment of Improvement Scale
GC-MS: Gas chromatography - mass spectrometry
G-CSF: Granulocyte colony stimulating factor
GM-CSF: Granulocyte-macrophage colony stimulating factor
GM: Gut microbiota
Gro-α: Growth-regulated oncogene-α
IBS: Inflammatory bowel syndrome
IFN-γ: Interferon gamma
IL-1ra: Interleukin-1ra
IL-4: Interleukin-4
IL-6: Interleukin-6
IL-8: Interleukin-8
IL-9: Interleukin-9
IL-10: Interleukin-10
IL-12: Interleukin-12
IL-17: Interleukin-17
IL-22: Interleukin-22
IP-10: IFN-γ-inducible protein 10
MCP-1: Monocyte chemoattractant protein-1
MD-Bt: Mediterranean diet supplemented with butyrate
MD: Mediterranean diet
MIP-1ß: Macrophage inflammatory protein-1ß
MS/MS: Tandem mass
OTU: Operational taxonomic unit
PCoA: Principal coordinates analysis
ROS: Reactive oxygen species
SCFA: Short-chain fatty acids
SIM: Single ion monitoring
SPME: Solid phase microextraction
SRM: Selected ion monitoring
SSS: Symptom severity scale
TAC: Total antioxidant capacity
TBARS: Thiobarbituric acid reactive substances
TGF-ß: Transforming growth factor beta
TNF-α: Tumor necrosis factor alpha
TIC: Total ion current scan
VAS: Visual analogue scale
VD: Lacto-ovo-vegetarian diet
VEGF: Vascular endothelial growth factor
