# Supplementary material for: Modulation of gut microbiota through nutritional interventions in Behçet’s syndrome patients (the MAMBA study): study protocol for a randomized controlled trial
Source: Trials. 2020 Jun 9;21:511. doi: 10.1186/s13063-020-04444-6 (PMC7282205; doi:10.1186/s13063-020-04444-6)
Supplement: Supplementary file 2 — Additional file 2. [file 13063_2020_4444_MOESM2_ESM.docx]

**MODULO DI CONSENSO INFORMATO**

Io sottoscritto/a ______________________________________________ nato/a il ___/___/______ residente a______________________ via/piazza ____________________________ Tel.________________ domicilio (se diverso dalla residenza) __________________________________

**DICHIARO**

- di aver ricevuto dal Dottor ………………………… esaurienti spiegazioni in merito alla richiesta di partecipazione alla ricerca in oggetto, secondo quanto riportato nella scheda informativa, facente parte di questo consenso, della quale mi è stata consegnata una copia in data ____________ alle ore ______________;
- che mi sono stati chiaramente spiegati e di aver compreso la natura, le finalità, le procedure, i benefici attesi, i rischi e gli inconvenienti possibili e le alternative dello studio clinico;
- di aver avuto l’opportunità di porre domande chiarificatrici e di aver avuto risposte soddisfacenti;
- di aver avuto tutto il tempo necessario prima di decidere se partecipare o meno;
- di non aver avuto alcuna coercizione indebita nella richiesta del Consenso;
- che mi è stato chiaramente spiegato di poter decidere liberamente di non prendere parte allo studio o di uscirne in qualsiasi momento senza fornire giustificazione, e che tali decisioni non modificheranno in alcun modo i rapporti con i medici curanti e con la struttura presso la quale sono in cura;
- di essere consapevole dell’importanza (e della mia responsabilità) di informare il mio medico di medicina generale della sperimentazione alla quale accetto di partecipare; nel caso decida di non informarlo, esonero sia il mio medico curante che i medici che mi seguono nella sperimentazione dalla responsabilità per i danni che possano derivare dall’incompatibilità tra il(i) farmaco(i) in studio ed altri trattamenti medici.

**DICHIARO pertanto di**

 **volere**  **NON volere**

partecipare allo studio

 **volere**  **NON volere**

essere informato sui risultati di questa ricerca dal medico dello studio

  **volere**  **NON volere**

essere informato sui risultati della ricerca dal medico dello studio, anche in relazione alle notizie inattese che dovessero essere accidentalmente riscontrate con le indagini previste dallo studio

 **volere**  **NON volere**

Informare il medico di medicina generale della partecipazione allo studio

_________________________________________ ___/___/______ _________ ________________

Nome per esteso del paziente (adulto, minore maturo) Data Ora Firma

__________________________________ ___/___/______ _________ ____________________

Nome per esteso rappresentante legale Data Ora Firma

|  |  |  |  |
| --- | --- | --- | --- |
| Io sottoscritto Dott. |  | ………………………………… | ………………………..… |
|  | | Cognome | Nome |
| Dichiaro che il Paziente ha firmato spontaneamente la sua partecipazione allo studio  Dichiaro inoltre di:   - aver fornito al Paziente esaurienti spiegazioni in merito alle finalità dello studio, alle procedure, ai possibili rischi e benefici e alle sue possibili alternative; - aver verificato che il Paziente abbia sufficientemente compreso le informazioni fornitegli - aver lasciato al Paziente il tempo necessario e la possibilità di fare domande in merito allo studio - non aver esercitato alcuna coercizione od influenza indebita nella richiesta del Consenso   ______________________________ ___/___/______ _________ ____________________  Nome per esteso del medico Data Ora Firma  che ha fornito le informazioni e  raccolto il consenso informato | | | |

| **NOTA BENE**  una copia del presente modulo, firmato e datato, allegato alle “Informazioni scritte per il Paziente” dovrà essere consegnata al Paziente stesso |
| --- |
